# Structural Characterisation of Tpx from *Yersinia pseudotuberculosis* Reveals Insights into the Binding of Salicylidene Acylhydrazide Compounds

**DOI:** 10.1371/journal.pone.0032217

**Published:** 2012-02-27

**Authors:** Mads Gabrielsen, Katherine S. H. Beckham, Victoria A. Feher, Caroline E. Zetterström, Dai Wang, Sylke Müller, Mikael Elofsson, Rommie E. Amaro, Olwyn Byron, Andrew J. Roe

**Affiliations:** 1 Institute of Infection, Immunity and Immunology, College of Medical, Veterinary and Life Sciences, University of Glasgow, Glasgow, United Kingdom; 2 Departments of Pharmaceutical Sciences, Computer Science, and Chemistry, University of California Irvine, Irvine, California, United States of America; 3 Department of Chemistry and Umeå Centre for Microbial Research, Umeå University, Umeå, Sweden; 4 Wellcome Centre for Molecular Parasitology, Institute of Infection, Immunity and Immunology, College of Medical, Veterinary and Life Sciences, University of Glasgow, Glasgow, United Kingdom; 5 School of Life Sciences, College of Medical, Veterinary and Life Sciences, University of Glasgow, Glasgow, United Kingdom; University of Oulu, Finland

## Abstract

Thiol peroxidase, Tpx, has been shown to be a target protein of the salicylidene acylhydrazide class of antivirulence compounds. In this study we present the crystal structures of Tpx from *Y. pseudotuberculosis* (*yp*Tpx) in the oxidised and reduced states, together with the structure of the C61S mutant. The structures solved are consistent with previously solved atypical 2-Cys thiol peroxidases, including that for “forced” reduced states using the C61S mutant. In addition, by investigating the solution structure of *yp*Tpx using small angle X-ray scattering (SAXS), we have confirmed that reduced state *yp*Tpx in solution is a homodimer. The solution structure also reveals flexibility around the dimer interface. Notably, the conformational changes observed between the redox states at the catalytic triad and at the dimer interface have implications for substrate and inhibitor binding. The structural data were used to model the binding of two salicylidene acylhydrazide compounds to the oxidised structure of *yp*Tpx. Overall, the study provides insights into the binding of the salicylidene acylhydrazides to *yp*Tpx, aiding our long-term strategy to understand the mode of action of this class of compounds.

## Introduction

**Figure 1:**
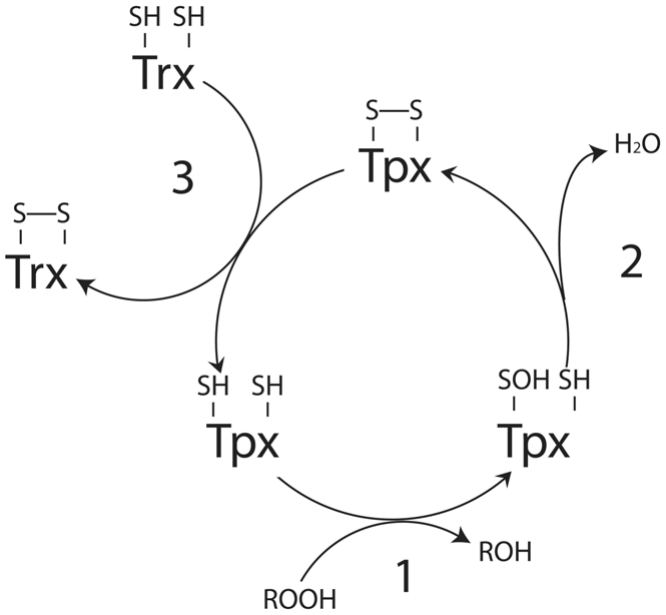
Redox cycle of Tpx. The reduction of ROS by oxidation (step 1) of Tpx producing H_2_O (step 2). The oxidised form (disulphide bond) of Tpx is recovered by thioredoxin (Trx), and returned to the reduced form (step 3).

Thiol peroxidase (Tpx, p20, scavengase) is an atypical 2-Cys peroxiredoxin present throughout the eubacteria, including pathogenic strains, such as *Escherichia coli* O157:H7 [1], *Yersinia* sp., *Haemophilus influenzae*, *Streptococcus pneumoniae* and *Helicobacter pylori* [2]. Tpx constitutes part of the bacterial defence system against reactive oxygen species (ROS) and, correspondingly, is upregulated when *E. coli* is exposed to oxidative stress [1]. Tpx functionality specifically relies on the reducing equivalents from thioredoxin (Trx1) and thioredoxin reductase (TrxR) [3]. The catalytic cycle of peroxiredoxin activity consists of three steps; 1) peroxidation, 2) resolution and 3) recycling [4]. Atypical 2-Cys peroxiredoxins are functionally monomeric, in contrast to the typical peroxiredoxins, i.e. the resolving (C_r_) and the peroxidasic (C_p_) cysteines (C61 and C95, respectively in the case of Tpx) are situated on the same subunit. Structurally, this involves the reduced Tpx encountering a ROS, such as hydrogen peroxide or an alkyl hydroperoxide, and the covalent binding of O^−^ to C_p_. The ROS is released as H_2_O resulting in the formation of a disulphide bridge between C_p_ and C_r_. The cycle is completed by a transient interaction with Trx1, ending with two separate cysteine side-chains on Tpx (Figure 1).

Tpx contains three cysteine residues, two of which (C61and C95) form the redox active disulphide bond. The third cysteine (C82) is not involved in the redox activities of Tpx [3], and is not involved in any covalent interactions. Until now, twelve structures of Tpx have been elucidated, from *E. coli* [4], [5], *Bacillus subtilis* [6], *Aquifex aeolicus*, *Mycobacterium tuberculosis* [7], [8], *H. influenzae*, and *S. pneumoniae*. Most of these structures have been solved in the oxidised state, or in the “forced” reduced state of the C61S (or equivalent) mutant. Two wild-type reduced structures have been solved, one by NMR [6] and one by X-ray crystallography (Structural Genomics Consortium).

Initially presumed to be localised in the periplasm [1], recent work using cross-linking and fractionation studies [9] has shown that Tpx is one of several peroxiredoxins in the cytosol of *E. coli*. Tpx has been shown to be important for the survival of *S. typhimurium* in macrophages, where the oxidative burst can be particularly acute [10].

We have recently shown that Tpx is one of several proteins bound by a class of “anti-virulence” compounds, the salicylidene acylhydrazides [11]. These compounds are broadly effective in reducing the expression of the type three secretion system (T3SS) of a range of Gram-negative pathogens including *Chlamydia* spp., *Salmonella enterica* serovar Typhimurium, *Yersinia pseudotuberculosis*, *Shigella flexneri*, and *E. coli* O157 [12]. The T3SS is a critical determinant used by pathogens to modulate host cell processes and facilitate processes such as binding and invasion [13] so compounds that interfere with its expression or function have the potential to become novel anti-infective agents [12], [14].

The precise molecular mechanism of action of the salicylidene acylhydrazides is not fully understood, although our identification of multiple binding proteins suggests a synergistic effect arising from a modulation of the activity of several proteins, including Tpx. The binding affinity of the salicylidene acylhydrazide compound ME0052 [N′-(3,5-dibromo-2-hydroxy-benzylidene)-nicotinic acid hydrazide] to Tpx from *Y. pseudotuberculosis* (*yp*Tpx) was measured using analytical ultracentrifugation (AUC) and showed that a catalytically inactive mutant of Tpx displayed a two-fold reduction in binding [11]. In this mutant, cysteine 61 was specifically mutated to a serine residue (C61S). The C61S mutant Tpx is present only in its reduced form and cannot undergo the intramolecular disulphide bond formation critical for the catalytic cycle of the protein.

In the current study, we present the crystal structures of *yp*Tpx in the oxidised and reduced states together with the structure of the C61S mutant. These structural data, combined with our previous NMR chemical shift analysis, allow us to perform detailed molecular modelling of how the salicylidene acylhydrazides bind to target proteins. This work helps our understanding of the mode-of-action of this class of anti-virulence compounds.

## Results and Discussion

### Purified Tpx is active and bound by the salicylidene acylhydrazides

**Figure 2:**
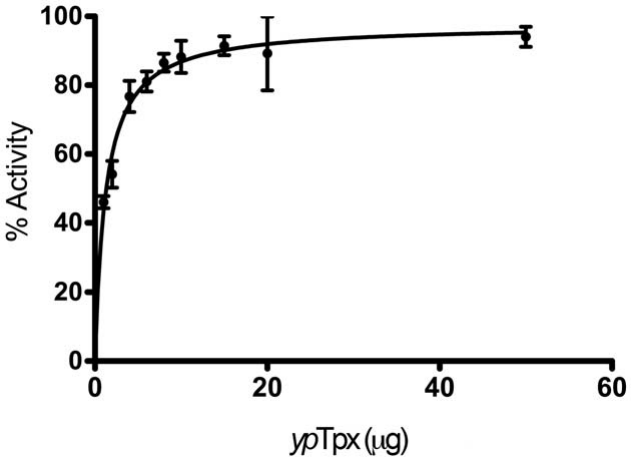
Glutamine synthetase assay. The activity of *yp*Tpx is confirmed by its ability to rescue the activity of glutamine syntethase (to 90% of original activity at 15 µg *yp*Tpx).

Tpx can be readily purified using nickel affinity chromatography, thereby facilitating structural and subsequent biochemical studies. The activity of purified *yp*Tpx was tested using a glutamine synthetase (GS) assay, where an active peroxiredoxin protects against ROS [15]. Two micrograms of *yp*Tpx rescued 50% of initial GS activity, with 15 µg of *yp*Tpx raising GS activity to 90% of initial activity, clearly demonstrating that *yp*Tpx was indeed active and reduced H_2_O_2_ (Figure 2). Previous characterisation of Tpx from *E. coli* (*ec*Tpx) has shown a substrate specificity for alkyl hydroperoxides over H_2_O_2_, with a *K_m_* of 9 µM for cumene peroxide compared with a *K_m_*>1.7 mM for H_2_O_2_ [3]. However, despite this lower substrate specificity, analysis of mutants in Salmonella revealed that a Tpx mutant was highly susceptible to exogenous H_2_O_2_ [10].

### Crystal structures

To determine the high-resolution structure of Tpx, recombinant *yp*Tpx in reduced and oxidised state, and the mutants *yp*TpxC61S were crystallised. *yp*Tpx crystallised in three crystal forms, 1, 2 and 3, in three different space groups, *P*2_1_, *P*6_4_ and *P*2_1_2_1_2_1_, respectively, as described elsewhere [16]. Crystal forms 1 and 2 grew in conditions containing DTT, and the solved structures were in the reduced state. Crystal form 1 diffracted to 2.00 Å, and the structure comprised three dimers in the asymmetric unit. The six chains superpose well, with root-mean-square-deviations (r.m.s.d.) of less than 0.5 Å. Crystal form 2, diffracting to 2.35 Å, comprises a single subunit in the asymmetric unit, the complete dimer being made up by symmetry operators.

As there are only minor differences between the two reduced structures, with an r.m.s.d. of 0.4 Å over 160 C_α_, only the highest resolution structure (space group *P*2_1_) will be discussed here. Most residues are accounted for in the electron density, apart from the hexa-histidine tag. The reduced structure refined to *R*_work_- and *R*_free_ -factors of 22.2% and 26.8%, respectively. Refinement statistics for all structures are presented in Table 1.

**Table 1:** Refinement statistics for reported structures.

	Reduced Tpx	Reduced Tpx	Oxidised Tpx	Tpx C61S
PDB code	2XPD	3ZRE	3ZRD	2YJH
Space group	*P*2_1_	*P*6_4_	*P*2_1_2_1_2_1_	*P*6_4_
Unit cell (Å)	a = 64.86b = 92.07c = 85.60β = 91.41°	a = 65.01c = 86.28	a = 56.18b = 62.56c = 88.00	a = 65.03c = 85.60
Resolution (Å)	44.67 – 2.00	56.30 - 2.35	28.09 - 1.74	56.32 - 2.55
Protein residues (atoms)	903 (7449)	165 (1191)	339 (2517)	164 (1188)
Water molecules	490	12	199	4
Ligands	DTT	N/A	N/A	N/A
R_work_ (%)	22.5	23.5	19.5	31.3
R_free_ (%)	26.8	27.3	23.13	33.6
R.m.s.d. for bond lengths (Å)/angles (°)	0.022/1.8	0.02/1.995	0.009/1.080	0.005/0.772
Ramachandran allowed regions (%)	99.8	97.6	100.0	97.6

**Figure 3:**
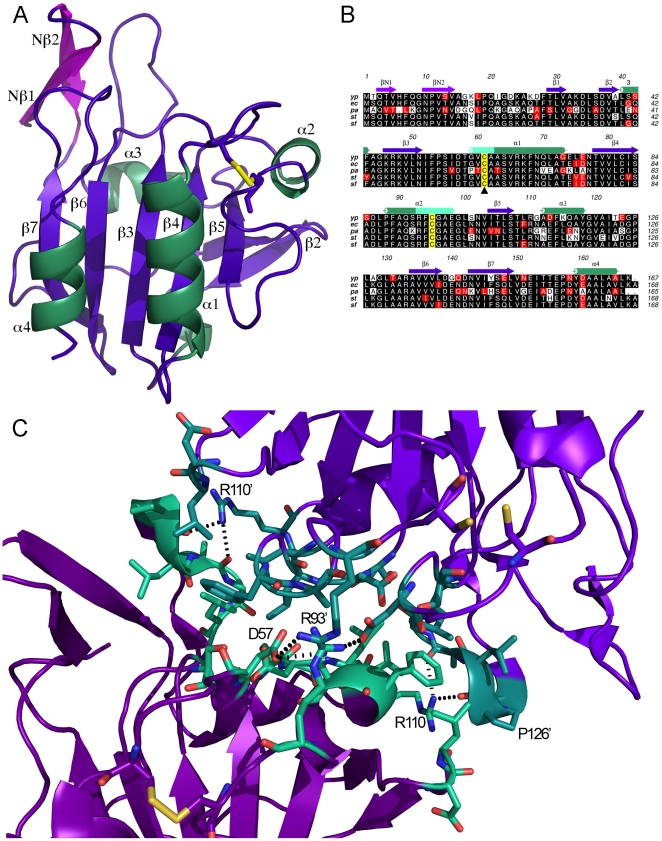
Crystal structures. (A) Cartoon representation of oxidised *yp*Tpx. Strands and loops are purple, helices are green, and the disulphide bond is represented as sticks. The N-terminal hairpin is highlighted in pink. The secondary structure elements are labelled. (B) Sequence alignment of Tpx from a number of pathogens, with the secondary structure based on *yp*Tpx. Black represents identical, and red highly similar residues (based on an ALSCRIPT algorithm level of 0.7 [53]). The unravelling regions of α1 and α2, caused by the change of redox state, are highlighted by the lighter colour in the secondary structure elements. The reactive C residues are highlighted in yellow, and C61S is marked by a triangle. (C) Close-up of the dimer interface with the residues involved interactions marked as sticks, and salt bridges and hydrogen bonding partners are labelled. Bonds are shown in dashes.

*yp*Tpx has a regular thioredoxin-like fold: a seven-stranded β-sheet, with β2 and β6 running anti-parallel to the rest, although with an inserted N-terminal β-hairpin (βN1-βN2) (Figure 3) absent from other peroxierdoxins. The central sheet is flanked by four α-helices following β3, β4, β5 and β7, and one short 3_10_ helix following β2. The numbering of the β-strands is based upon that of *ec*Tpx [5] to make direct comparisons between all Tpx molecules easier. βN1-βN2 (Figure 3) forms an L-shaped hydrophobic cleft, and it has been speculated that this cleft allows Tpx to accommodate the long fatty acid hydroperoxides [17].

Crystal form 3 captured *yp*Tpx in the oxidised state with an intact intramolecular disulphide bond between Cys61 and Cys95. The crystal belonged to space group *P*2_1_2_1_2_1_ and diffracted to 1.74 Å. The overall oxidised structure, diffracting to a resolution of 1.74 Å and presenting space group *P*2_1_2_1_2_1_, is similar to that of the reduced structure, except for some differences that are mostly confined to the region around the active site (see below).

As part of this study, the structure of *yp*TpxC61S was solved to a resolution of 2.55 Å in space group *P*6_4_. This structure represents the “forced” reduced form of the protein, as the resolving cysteine has been mutated to a serine, rendering it catalytically inactive [3]. All of our solution data indicate that the mutant structure and the reduced wild type structure are identical, and that the oligomeric states are the same. When superimposed onto the reduced structure, the r.m.s.d. was 0.52 Å over 163 C_α_ (Figure S1). This fits well with the structural analysis of Hall et al. [4] who used the TpxC61S mutant from *E. coli* to describe the structure of reduced Tpx.

*yp*Tpx crystallised either as a dimer in the asymmetric unit, where the two subunits superposed with an r.m.s.d. of less than 0.2 Å, or the dimer could be created by crystallographic symmetry operators. The dimer interface comprises about 20 residues from each subunit, corresponding to 12% of total surface residues, according to the PISA server [18]. The interfaces are formed mostly by hydrophobic interactions, with a few hydrogen bonds, namely between R110NH1 and three main-chain carboxyl groups on the opposing subunit (G125, P126, A128). There are no salt bridges or covalent bonds between the two dimers in the reduced structure. The dimer interfaces are identical in the structures of reduced Tpx and C61S.

The dimer interface is of similar size in the oxidised state but in addition to the hydrogen bond pattern described above, there are also salt bridges formed between D57 and R93 on opposing subunits (Figure 3C). This is due to the conformational change between the reduced and oxidised states.

### Active site

**Figure 4:**
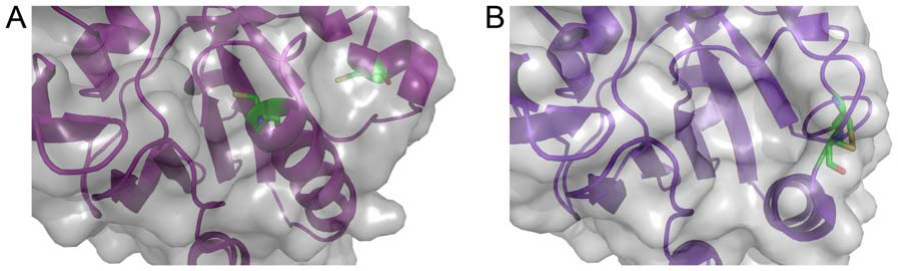
Comparison of the oxidised and reduced active site. (A) Close-up of the active cysteines in the reduced structure. C61 is shown to occlude the active site cleft. (B) Close-up of the reduced structure. The formation of the disulphide bond shifts the helix and opens a cleft, which allows substrate access.

The redox active site of Tpx is made up by C_P_ and C_R_ (C61 and C95 in *yp*Tpx, respectively). There is a conformational change between the two states, involving the partial unfolding of helices α1 and α2, and a shift of 8.4 Å for C61 and 5.1 Å for C95, respectively, as presented in Figure 4. The two structures superpose well, in particular the core parts, with an r.m.s.d of 0.7 Å over 135 C_α_. Inclusion of the unfolding helices increases the r.m.s.d. to 1.02 Å.

The partial unfolding of α1 opens a cleft in Tpx formed between the loops connecting β1 and α1, α3 and β6, and β7 and α5 on subunit A and connecting β1 and β2, and β4 and α2 on subunit B. When in the reduced state, C61 is orientated into the pocket where it is available for oxidation by H_2_O_2_ or alkyl peroxides. This cleft makes up the active site of Tpx, and has been described in detail by Hall et al. [4]. In this manuscript they present the fully intact peroxide binding site (a reduced C61S mutant), the locally unfolded binding site (oxidised), and a partially unfolded transitional state (seen only for the double C82, 95S mutant) for Tpx from *E. coli* that shares an identical active site.

### Oligomeric state

**Figure 5:**
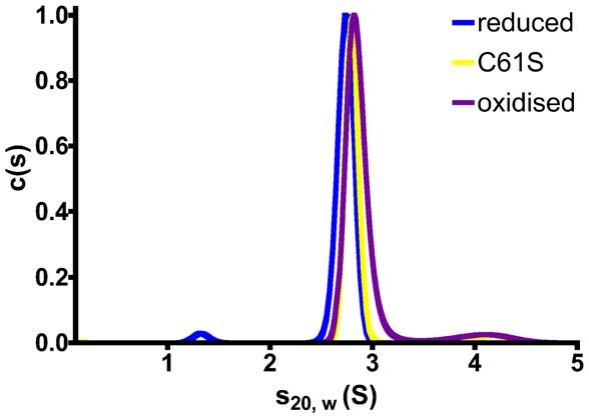
Analytical ultracentrifugation. c(s) distributions derived via SEDFIT from SV data for *yp*Tpx in oxidised (purple) and reduced (blue) states, as well as *yp*TpxC61S (yellow) indicate that the protein is monodisperse (dominated by a single, symmetrical peak) with the same sedimentation coefficient regardless of redox state.

Peroxiredoxins exhibit a wide variety of oligomeric states, ranging from monomeric (YPrx, [19]), to large decameric or dodecameric assemblies like TryP [20], AhpC [21] and other typical 2-Cys peroxiredoxins, including PrxIII from bovine mitochondria, which forms two concatenated dodecamers [22]. These assemblies are often dependent on redox state, dissociating into homodimers upon oxidation [23]. Previous studies of *ec*Tpx showed that the protein is a homodimer, regardless of the redox state, and despite the lack of any inter-subunit disulphide bond [3]. We analysed the oligomeric state of oxidised and reduced *yp*Tpx, as well as the C61S mutant by AUC and SAXS. Sedimentation velocity (SV) experiments revealed that all three forms of *yp*Tpx were completely monodisperse in solution, as evidenced by a single dominant peak in the c(s) distribution (Figure 5). Infinite dilution sedimentation coefficients (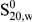) were determined from the concentration dependence of s_20,w_ (obtained from fitting the SV data with a non-interacting discrete species model in SEDFIT [24]) for the oxidised and reduced forms of *yp*Tpx (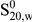 = 3.04 and 2.62 S, respectively). For *yp*Tpx C61S 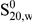 is 2.80 S, suggesting that this mutation does not induce structural instability (i.e. the value is comparable with those determined for reduced and oxidised *yp*Tpx).

Sedimentation equilibrium (SE) data were fitted with the species analysis model in SEDPHAT [25]. From the concentration dependence of the resultant apparent mass of the single species the infinite dilution mass (M^0^) was determined to be 41.5±3.4, 38.4±2.9, 39.4±0.4 kDa for oxidised, reduced and C61S *yp*Tpx respectively. The mass of *yp*Tpx dimer, including the tag, calculated from its amino acid sequence is 42,382 Da, which is consistent with the experimentally determined masses. This indicates that *yp*Tpx is present solely as a dimer in solution. It was not possible to fit the SE data with a monomer-dimer (or any other plausible) self-association model, which is further consistent with the complete dimerisation of the protein in the concentration range studied.

### Solution structure

**Figure 6:**
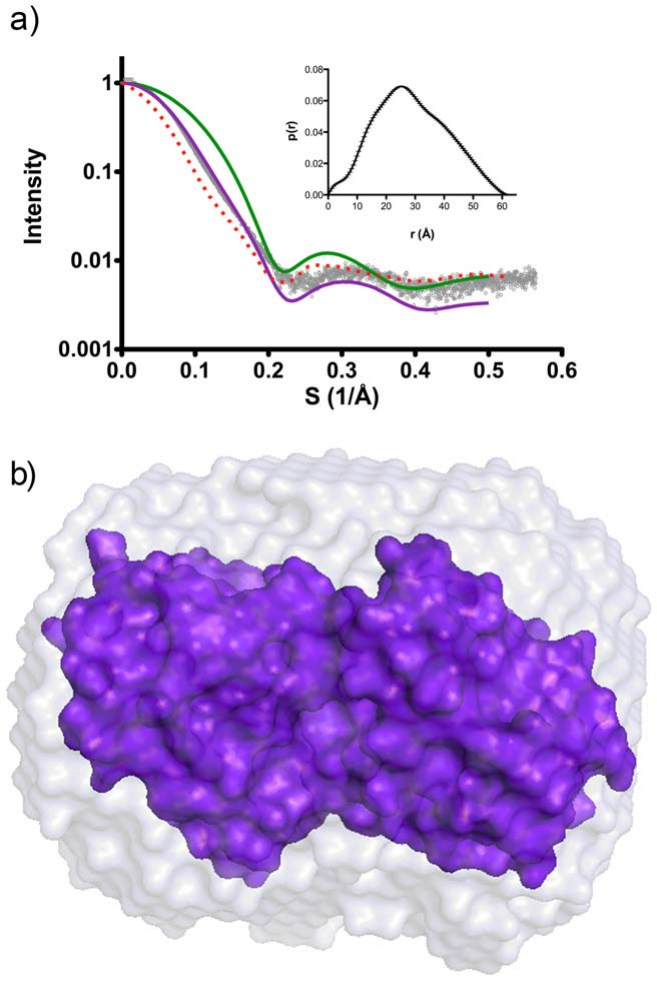
SAXS results. (A) The experimental scattering curve of *yp*TpxC61S (grey), overlaid with the curves for monomeric Tpx (green) and dimeric Tpx (purple) (calculated using CRYSOL [50]) confirming the dimeric solution state of the protein. The fit of the *ab initio* model to the experimental data is shown in red. Inset is the distance distribution function (p(r) *versus* r) of *yp*TpxC61S with error bars. (B) Space-filled crystal structure of *yp*TpxC61S (purple) superposed onto the averaged DAMMIN *ab initio* model (grey) using the program SUPCOMB [49].

The solution structures of *yp*Tpx and the C61S mutant were investigated using SAXS, a powerful method to structurally analyse proteins in solution under more physiologically relevant conditions [26]. Figure 6A shows a SAXS curve for *yp*TpxC61S, representative of the data obtained for *yp*Tpx in both oxidising and reducing conditions. *yp*TpxC61S was obtained at a higher concentration than the other samples, and subsequently produced better scattering data. The D_max_ and R_g_ of *yp*Tpx and *yp*TpxC61S, obtained by indirect Fourier transform with GNOM [27], were the same (70.5 Å and 24.0±0.2 Å, respectively) indicative that conformational changes induced by disulphide bond formation are too small to be detected by SAXS. Theoretical scattering curves of monomeric and dimeric atomic structures of Tpx were calculated, and again confirm that Tpx is a dimer in solution (Figure 6A). A low-resolution (11 Å) envelope of *yp*TpxC61S (Figure 6B) in solution was generated using the *ab initio* modelling program DAMMIN [28]. The fit of the model to the data is shown in Figure 6A.

The high-resolution structure superimposes well onto the low-resolution envelope (Figure 6B). The D_max_ of the space-fill model of the dimer crystal structure is approximately 68 Å, which agrees with the D_max_ obtained from the SAXS data (70.5 Å), indicating that the low-resolution envelope describes the *yp*Tpx dimer. The differences in the D_max_ values obtained from the two methods are small, and may be explained by the fact that in the crystal structure there is no electron density to account for the two N-terminal residues of Tpx plus the hexa-histidine tag, therefore it has not been included in calculations. However, as these residues were present in the *yp*Tpx studied by SAXS, we would expect the D_max_ value observed in solution by SAXS to exceed that calculated for the incomplete crystal structure.

Rigid body modelling of the oxidised Tpx crystal structure against the SAXS data, using BUNCH [29], based on a single chain, and imposing *P*2 symmetry yielded a model similar to that for the dimeric crystal structure. Comparison of the crystallographic model with the one fitted to the solution data using DYNDOM [30] yielded a rotation angle of 21.4° and a 5 Å translation. This freedom of movement corresponds well with that observed for the structures of Tpx from other different species [4].

### Modelling of salicylidene acylhydrazide compounds to *yp*Tpx

**Figure 7:**
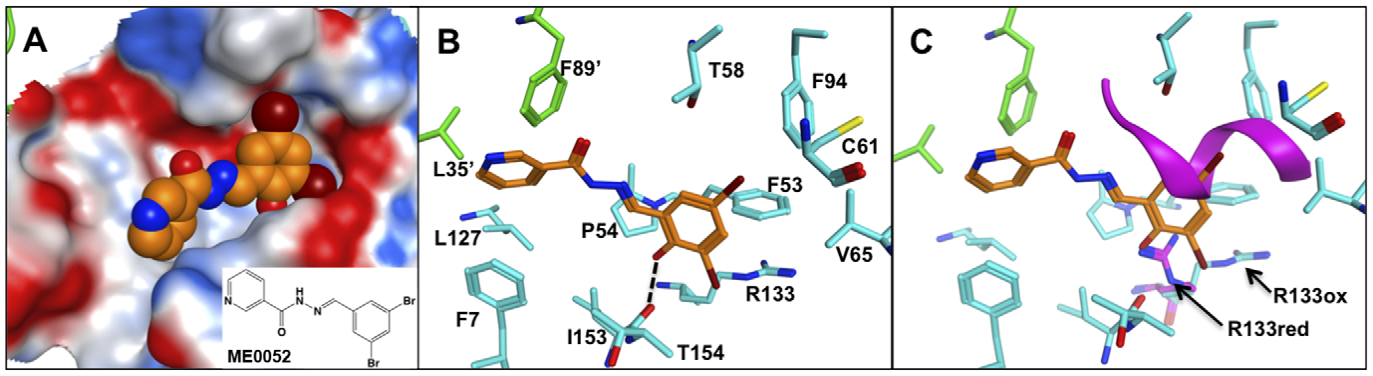
Model for salicylidene acylhyrazide ME0052 binding mode to oxidised *yp*Tpx crystal structure. (A) Binding mode for lowest docked energy ME0052 (atoms represented as CPK orange for carbon, blue for nitrogen and red for bromines) and electrostatic surface for the protein binding site (Connolly surface, sphere radius = 1.4 Å). ChemDraw representation for the ME0052 inhibitor is inset. (B) Detailed view of ME0052 binding mode (stick representation). Oxidised *yp*Tpx residues with atom contacts within 3.5 Å of ME0052 inhibitor are shown (cyan). Residues F89′ and L35′ are contributed by the neighbouring monomer (green). A hydrogen bond (dashed line) is formed between inhibitor and I153 carbonyl. (C) ME0052 binding mode from (B) with reduced *yp*Tpx (protein backbone magenta ribbon) superposed on oxidised *yp*Tpx; shown are α1 helix and the R133 side chain of the reduced form partially occluding the binding pocket of oxidised form.

We have previously used NMR chemical shift mapping to identify *yp*Tpx amide groups that were shifted upon addition of 200 µM ME0052. The study mapped these shifting residues onto the published TpxC61S structure from *E. coli* (PDB code 3HVV) to show they clustered to a defined region of the protein. Now we have obtained the high-resolution structure of *yp*Tpx itself, allowing us to model the binding of ME0052 and ME0055 to both the oxidised and reduced forms of the protein and examine how this correlates with the NMR data. These two compounds were docked into the receptor structures using MOE Dock, and the 25 best poses determined for each compound were ranked after energy minimisation and dock scoring. Figure 7 shows the lowest energy binding modes for ME0052 (Figure 7A) bound to oxidised *yp*Tpx. Docking using ME0055 gave equivalent binding poses (data not shown). The binding pocket is mostly hydrophobic (Figure 7A) with one hydrogen bond proposed between the ME0052 p-hydroxyl to the *yp*Tpx I153 carbonyl, which fits with the chemical shift change for the neighbouring T154 amide (Figure 7B). The chemical shift data indicated significant shifts in the amides of residues from both subunits, highlighting the importance of the dimer interface for the generation of the binding pocket and compound binding, as illustrated in Figure S2A. The binding site is also consistent with previously published Tpx-substrate models [32].

During transition to the reduced form, the loop containing G59 to C61 folds into the binding pocket predicted to accommodate the compound. Similarly, the side chain of R133 undergoes a significant conformational change. Collectively, these conformational changes reduce the overall volume of the binding pocket (Figure 7C) and are predicted to affect the binding of ME0052 to the reduced state of *yp*Tpx by inducing steric clashes. In fact, MOE Dock as not able to find a suitable docking pose for the reduced state of *yp*Tpx, presumably because the binding site is occluded by the folded extension of the α1 helix in the reduced state.

Accurately measuring the binding of the salicylidene acylhydrazides to any protein has proved problematic due to the low solubility of the compounds in physiologically relevant solvents. This has prohibited the application of techniques including isothermal calorimetery and surface plasmon resonance that would be default methods to measure binding of ligands to proteins. Previously we have used an AUC-based method to estimate K_d_, giving values of 51 and 71 µM for the binding of ME0052 to oxidised and reduced *yp*Tpx, respectively [11]. These data suggest only minor differences in compound binding to *yp*Tpx in the two oxidation states, although we would exercise caution in the interpretation of these data due to the inherent inaccuracy of measuring K_d_ by this method.

An alternative binding pose, that would accommodate binding of ME0052 or ME0055, to both the oxidised and reduced forms of *yp*Tpx is presented as Figure S2B Although this model would require some conformational accommodation of the binding site, the compounds are predicted to be less buried in the pocket and therefore binding would be largely equivalent irrespective of the oxidation state of Tpx. Such conformational accommodation is plausible given the large backbone fluctuations observed in each state.

### Far-western analysis

**Figure 8:**
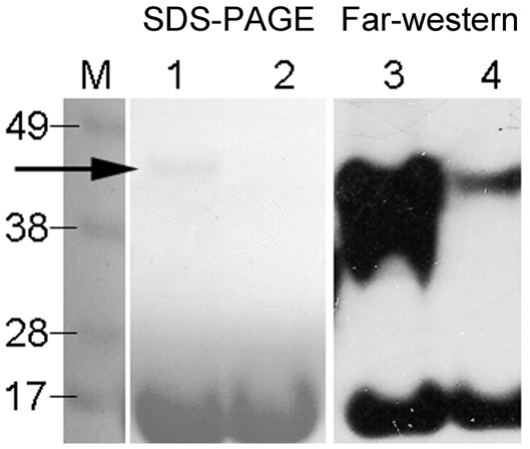
Far-western of purified *yp*Tpx. SDS-PAGE and Coomassie staining of: lane M, molecular weight ladder; lane 1, *yp*Tpx; lane 2, *yp*TpxC61S and (lanes 3 and 4 respectively) far-western of: *yp*Tpx and *yp*TpxC61S probed with ME0052-bio. The arrow highlights the presence of a small amount of dimeric *yp*Tpx on the SDS-PAGE, which can be contrasted with the far stronger signal from the corresponding band on the far-western.

To test the binding of *yp*Tpx to salicylidene acylhydrazide compounds, far-western blotting was used in which the protein was resolved by SDS-PAGE, transferred to a nitrocellulose membrane and probed using biotinylated ME0052 (ME0052-bio) [33], [34]. Interactions were then detected using a StreptAvidin-HRP conjugate in a process similar to that used for a routine western blot. Following SDS-PAGE and staining by Coomassie blue, Tpx could be seen as a monomer of 21 kDa as well as a dimer of 42 kDa (Figure 8). In comparison, the purified C61S mutant was present only as a monomer of 21 kDa. This difference can be attributed to the changes in the strength of the dimer interface, arising from the loss of two salt bridges in the ‘forced’ reduced C61S mutant, which in turn is more susceptible to the heat and detergent experienced during the far-western blotting procedure. Far-western analysis indicated that ME0052-bio binds with a far higher affinity to *yp*Tpx dimer, as over four times the amount of signal was seen corresponding to the dimer compared with the monomer (see Material and Methods). This finding is particularly stark when the relative proportions of dimer and monomer indicated on the gel are considered: quantification of the monomer-dimer on the SDS-PAGE gel indicated the ratio of these two species was 15∶1. This finding confirmed our previous data [11] showing binding of ME0052-bio when tested against *E. coli* overexpressing *ec*Tpx but is more unambiguous as it demonstrates binding to purified protein rather than through probing crude lysates. The caveat with this approach is that the protein must refold before binding the ligand. It could be that the better binding of the ligand to the dimer is simply due to an inherently more stable dimeric structure compared with the dissociated monomer. Therefore, the dimer refolds better than the monomer and gives more signal. However, despite this reservation, the preferential binding of the compound to the *yp*Tpx dimer is consistent with our modelling data and previous NMR studies that indicated both subunits contribute to the binding pocket [11].

### Conclusion

In summary, we have solved the high-resolution structure of *yp*Tpx in three forms enabling us to model the binding of salicylidene acylhydrazide compounds. Binding of the compound, ME0052, was found (by far-western blotting) to be markedly stronger to the Tpx dimer compared with the monomer. This is consistent with the surface area of the modelled binding site predominantly comprising one subunit yet also including the dimer interface. The solution structure confirms the oligomeric state of the protein for both redox states.

Overall, the study provides insights into the binding of the salicylidene acylhydrazide compounds to *yp*Tpx, aiding our long-term strategy aiming to understand the mode of action of these compounds. Further studies characterising the role of the conformational flexibility observed around the catalytic triad and dimeric interface in ligand binding may yield additional insights into the binding mechanisms of these compounds and guide efforts to design even more effective inhibitors.

## Materials and Methods

### Protein expression and purification

*yp*Tpx and *yp*TpxC61S were expressed and purified as described previously [16], and the N-terminal hexa-histidine tag formed part of the expressed protein.

### Glutamine synthetase assay

The enzymatic activity of *yp*Tpx was demonstrated by a glutamine synthetase (GS) protection assay [15]. Briefly, 4 µl (6.7 U) of commercially purchased GS (Sigma) was mixed with increasing amounts of *yp*Tpx (1 µg, 2 µg, 4 µg, 6 µg, 8 µg, 10 µg, 15 µg, 20 µg and 50 µg) and 10 µl inactivation solution (50 mM DTT, 25 µM FeCl_3_), in a final volume of 100 µl (made up in 100 mM HEPES pH 7.4). The mix was incubated for 30 min at room temperature. 2 ml of assay mix (100 mM HEPES, 10 mM KH_2_AsO_4_, 20 mM NH_2_OH, 0.4 mM ADP, 0.5 mM MnCl_2_, 100 mM glutamine, pH 7.0–7.2) was added to each solution and the incubation continued at 37°C. After 30 min, 1 ml of stop solution (5.5% (w/v) FeCl_3_, 2% (w/v) TCA, 2.1% (v/v) concentrated HCl) terminated the reaction. Absorbance of the samples was measured at 540 nm.

### Protein crystallisation

Purified proteins were dialysed overnight against 20 mM Tris pH 7.5, 50 mM NaCl and kept at a concentration of approximately 8 mg ml^−1^ (based on the absorbance at 280 nm, and a calculated extinction coefficient of 4595 M^−1^ cm^−1^), for crystallisation studies using crystallisation conditions described previously [16].

### Diffraction data collection and structure solution

All diffraction data were collected at Diamond Light Source (Oxfordshire UK), processed with MOSFLM [35] and scaled in SCALA [36], both parts of the CCP4 suite of programs [37], [38], or d*TREK [39]. The relevant statistics are published elsewhere [16]. An improved data set for the oxidised structure of *yp*Tpx was collected and the relevant statistics are found in Table S1.

The structure of *E. coli* Tpx (PDB 3HVV) was used to solve the structures of *yp*Tpx by molecular replacement using PHASER [40] as described previously [16]. Models were refined using REFMAC5 [41] and BUSTER [42], using TLS parameterisation, and inspected, and manipulated when required, in COOT [43], where waters were added. Models were validated in COOT and by the MolProbity server [44]. PDB files were superimposed using LSQMAN [45].

### Analytical ultracentrifugation

AUC was carried out in a Beckman Coulter (Palo Alto, CA) Optima XL-I analytical ultracentrifuge. Sedimentation velocity (SV) experiments were performed at 4°C at a rotor speed of 49 k rpm. 360 µl of *yp*Tpx or *yp*TpxC61S in 20 mM Tris pH 7.5, 50 mM NaCl, at four different concentrations between 0.2 and 20 mg ml^−1^, were loaded into double sector centrepieces. To impose oxidising or reducing conditions, 10 mM H_2_O_2_ or 5 mM DTT, respectively, were added to the samples. Data were acquired with interference optics; scans were taken every 7 minutes. Data were analysed using SEDFIT [24]. The partial specific volume of *yp*Tpx (0.7407 g ml^−1^/0.7138 g ml^−1^) and the buffer density (1.00264 g ml^−1^/1.00100 g ml^−1^) and viscosity (0.015835 P/0.010126 P) at 4°C and 20°C respectively, were calculated using the program SEDNTERP [46]. Sedimentation equilibrium (SE) experiments were performed at 4°C and at rotor speeds of 18 and 24 k rpm. Samples of 80 µl were loaded under the same conditions as for the SV experiments. Scans were taken every 3 h until analysis of the scans with WinMATCH (Jeffrey Lary, University of Connecticut, Storrs, CT, USA) indicated that equilibrium had been reached. SE data were analysed with SEDPHAT [25].

### Small angle X-ray scattering (SAXS)

SAXS data were collected on the EMBL ×33 beamline at the DORIS storage ring of the DESY (Deutsches Elektronen Synchrotron) synchrotron (Hamburg, Germany). X-rays were scattered from samples of varying concentrations of *yp*Tpx or *yp*TpxC61S in 20 mM Tris pH 7.5, 100 mM NaCl. Experiments were carried out at 4°C. Data were processed using the program PRIMUS [47]. The distance distribution function and maximum particle dimension (D_max_) were determined using the program GNOM [27] (Part of ATSAS 2.4 program suite, EMBL Hamburg). *Ab initio* modelling of *yp*Tpx was carried out using the program DAMMIN [28]. Twenty DAMMIN models were generated with an imposed 2-fold (*P*2) symmetry and were merged and averaged using the DAMAVER program suite [48]. The averaged DAMMIN model was superimposed onto the *yp*Tpx dimer crystal structure using SUPCOMB [49]. Theoretical scattering curves of the *yp*Tpx monomer and dimer were generated from the crystal structure coordinates using CRYSOL [50].

Crystal structures were modelled against the solution structure data by rigid body fitting, using BUNCH [29]. In order to ensure maintenance of the correct dimer interface in the reconstruction, distance restraints between interacting interface residues were imposed, namely a maximum distance of 7 Å between D57 and R93, and 4 Å between R110 and G125 (based on data from the high-resolution structure).

### Modelling of binding between Tpx and anti-virulence compounds

All modeling was performed using Molecular Operating Environment (MOE) (Chemical Computing Group Inc., Montreal, Canada) software. Modeled ME0052 and ME0055 were subjected to conformational searches for their lowest energy conformations as docking input. The structures of oxidised and reduced *yp*Tpx were subjected to energy minimisation with the MMFF94 force field and the GBSA solvation model prior to docking. MOE Dock (MOE 2010 version 2010.10) was used in the alpha triangle mode; the 25 best poses retained for each compound based on the Affinity dG MOE dock score were further ranked after energy minimisation.

Figures were made using PyMOL (www.pymol.org), ALINE [51], and MOE. All crystal structures have been deposited with the Protein Data Bank (codes presented in Table 1).

### Far-western blotting

Samples of *yp*Tpx and *yp*TpxC61S (100 µM) were heated for 10 min at 95°C with LDS loading buffer (NU-PAGE, Invitrogen) and run in MES buffer (NU-PAGE, Invitrogen) on a 4–12% Bis-Tris Novex gel (Invitrogen). The samples were blotted onto a nitrocelluose membrane and the far-western blot was carried out as described previously [11], [52]. Briefly, the blot was incubated for 4 h at RT in 5% skimmed milk and was probed with 2 µM ME0052-bio in PBS-Tween for 16 h at 4°C; biotin was detected using HRP-conjugated StreptAvidin (Invitrogen). Band intensities were quantified using ImageJ software (Rasband, http://imagej.nih.gov/ij/, 1997–2011).

## Supporting Information

Figure S1 **The structure of** ***yp*** **TpxC61S superposed onto that for** ***yp*** **Tpx in the reduced state with an r.m.s.d. of 0.7 Å indicating that the overall fold of the proteins is highly conserved.**
(TIF)

Figure S2 (A) Model for salicylidene acylhydrazide ME0052 (CPK representation) binding to oxidised *yp*Tpx in lowest energy docked conformation (backbone ribbon representation, Connolly surface of sphere radius 1.4 Å). Each subunit of the homodimer is coloured differently (cyan and green ribbon). Spheres on the protein ribbon represent amide groups with largest (blue) and moderate (yellow) chemical shift perturbation as judged by NMR HSQC when ME0052 binds [31]. *Amide sphere for L127 (blue). (B) Alternate binding mode for ME0052 (CPK) to oxidised *yp*Tpx (cyan ribbon) with reduced *yp*Tpx backbone superposed (magenta ribbon). The arrow points to the region of significant backbone and α1 conformational change between oxidised and reduced states.
(TIF)

Table S1 Data collection statistics for the oxidized *yp*Tpx obtained in this study. Values in brackets denote highest resolution shell.
(DOCX)
