# Supplementary figures and images for: Structural Characterisation of Tpx from Yersinia pseudotuberculosis Reveals Insights into the Binding of Salicylidene Acylhydrazide Compounds
Source: PLoS One. 2012 Feb 27;7(2):e32217. doi: 10.1371/journal.pone.0032217 (PMC3288085; doi:10.1371/journal.pone.0032217)

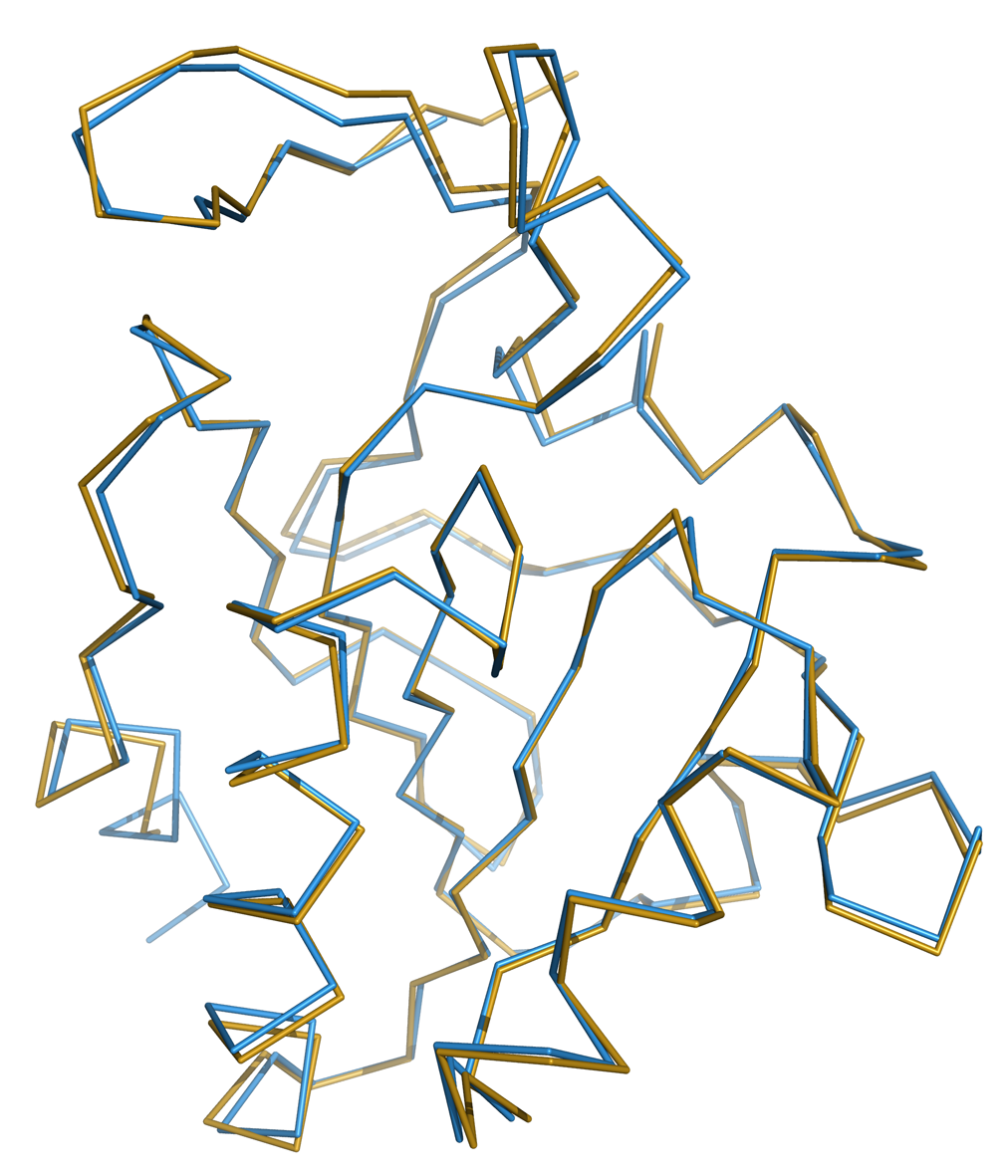

Supplement: Figure S1 — The structure of yp TpxC61S superposed onto that for yp Tpx in the reduced state with an r.m.s.d. of 0.7 Å indicating that the overall fold of the proteins is highly conserved. (TIF) [file pone.0032217.s001.tif]

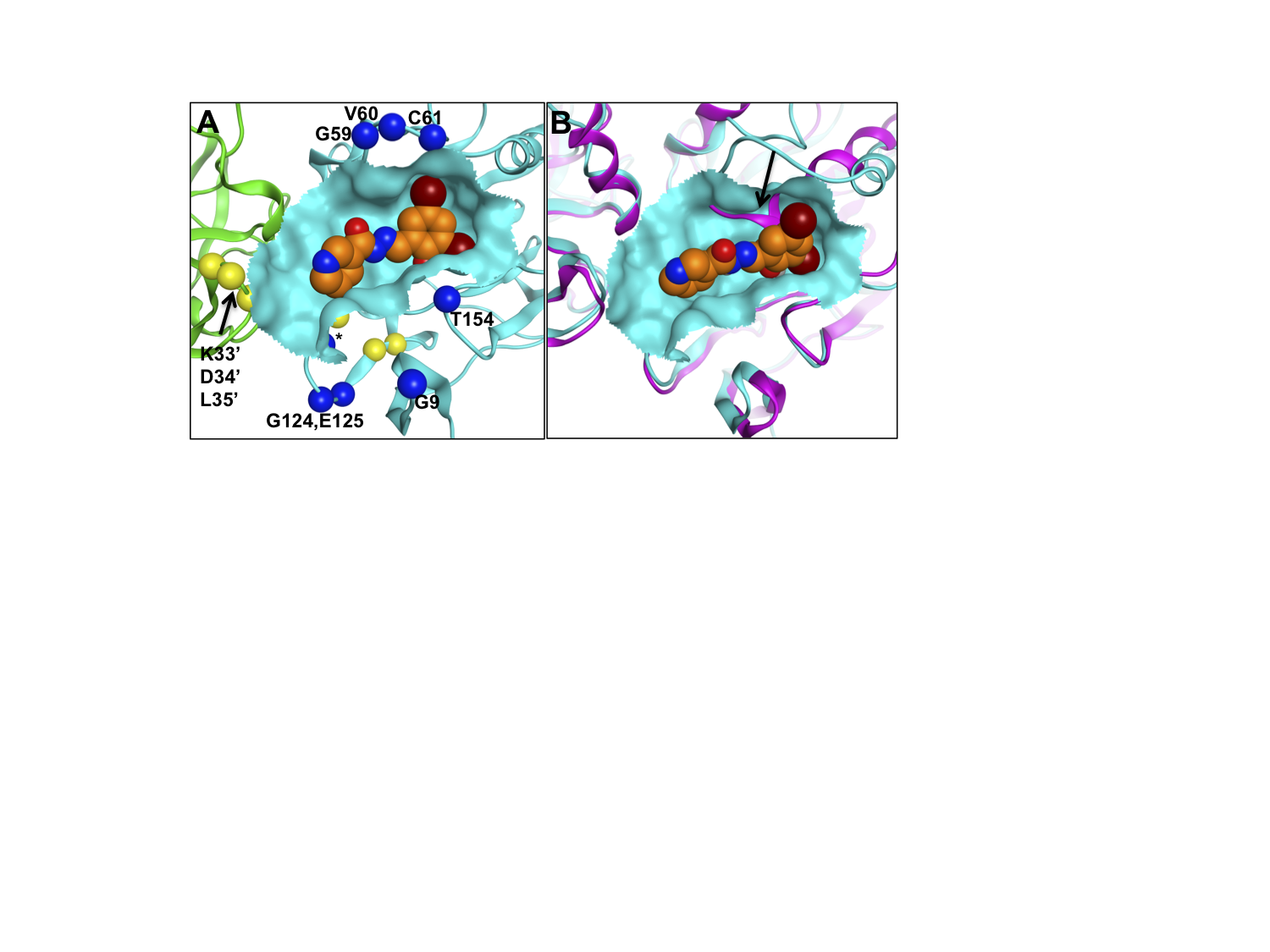

Supplement: Figure S2 — (A) Model for salicylidene acylhydrazide ME0052 (CPK representation) binding to oxidised ypTpx in lowest energy docked conformation (backbone ribbon representation, Connolly surface of sphere radius 1.4 Å). Each subunit of the homodimer is coloured differently (cyan and green ribbon). Spheres on the protein ribbon represent amide groups with largest (blue) and moderate (yellow) chemical shift perturbation as judged by NMR HSQC when ME0052 binds [31]. *Amide sphere for L127 (blue). (B) Alternate binding mode for ME0052 (CPK) to oxidised ypTpx (cyan ribbon) with reduced ypTpx backbone superposed (magenta ribbon). The arrow points to the region of significant backbone and α1 conformational change between oxidised and reduced states. (TIF) [file pone.0032217.s002.tif]
