# Supplementary material for: Structural Characterisation of Tpx from Yersinia pseudotuberculosis Reveals Insights into the Binding of Salicylidene Acylhydrazide Compounds
Source: PLoS One. 2012 Feb 27;7(2):e32217. doi: 10.1371/journal.pone.0032217 (PMC3288085; doi:10.1371/journal.pone.0032217)
Supplement: Table S1 — Data collection statistics for the oxidized ypTpx obtained in this study. Values in brackets denote highest resolution shell. (DOCX) [file pone.0032217.s003.docx]

Table S1:

| Crystal Form | Crystal Form Ox |
| --- | --- |
| Space group | *P*2_1_2_1_2_1_ |
| Unit Cell (Å) | *a*= 56.19  *b*= 62.55  *c*= 87.97 |
| Resolution (Å) | 56.19 - 1.98 (2.09 - 1.98) |
| Observed reflections | 86523 |
| Unique reflections | 22331 |
| Multiplicity | 3.9 (3.9) |
| Completeness (%) | 99.9 (100) |
| Matthews (Å^3^ Da^-1^) | 2.24 |
| Solvent content (%) | 45.20 |
| Monomers in asymmetric unit | 2 |
| *R*_meas_ (%) | 7.7 (25.5) |
| *R*_pim_ (%) | 3.9 (12.9) |
| I/σI | 13.8 (5.7) |
| Wilson B (Å^2^) | 21.1 |
